# Variations in the estimated intake of acrylamide from food in the Japanese population

**DOI:** 10.1186/s12937-020-00534-y

**Published:** 2020-02-21

**Authors:** Kumiko Kito, Junko Ishihara, Junpei Yamamoto, Takayuki Hosoda, Ayaka Kotemori, Ribeka Takachi, Kazutoshi Nakamura, Junta Tanaka, Taiki Yamaji, Taichi Shimazu, Yuri Ishii, Norie Sawada, Motoki Iwasaki, Hiroyasu Iso, Tomotaka Sobue, Shoichiro Tsugane

**Affiliations:** 1 Graduate School of Environmental Health, Azabu University, 1-17-71 Fuchinobe, Chuo-ku, Sagamihara-city, Kanagawa 252-5201 Japan; 2 Department of Food and Life Science, Azabu University, 1-17-71 Fuchinobe, Chuo-ku, Sagamihara-city, Kanagawa 252-5201 Japan; 3 Department of Food Science and Nutrition, Nara Women’s University Graduate School of Humanities and Sciences, Kitauoyahigashimachi Nara-shi, Nara, 630-8506 Japan; 4 Division of Preventive Medicine, Niigata University Graduate School of Medical and Dental Sciences, 1-757 Asahimachidori, Niigata, 951-8510 Japan; 5 Department of Health Promotion Medicine, Niigata University Graduate School of Medical and Dental Sciences, 1-757 Asahimachidori, Niigata, 951-8510 Japan; 6 Epidemiology and Prevention Group, Center for Public Health Sciences, National Cancer Center, 5-1-1 Tsukiji, Chuo-ku, Tokyo, 104-0045 Japan; 7 Public Health, Department of Social Medicine, Graduate School of Medicine, Osaka University, 2-2 Yamadaoka, Suita-city, Osaka 565-0871 Japan; 8 Department of Environmental Medicine and Population Sciences, Graduate School of Medicine, Osaka University, 2-2 Yamadaoka, Suita-city, Osaka 565-0871 Japan

**Keywords:** Acrylamide, Variation, Validity, Food frequency questionnaire, Dietary record

## Abstract

**Background:**

Due to concerns of carcinogenicity, it is necessary to assess long-term acrylamide exposure in individuals. Whether the available methods of estimating acrylamide intake can indicate long-term exposure remains unknown. We examined variations in the estimated dietary acrylamide intake of the Japanese population.

**Methods:**

The study included 240 participants aged 40–74 years who were a part of the Japan Public Health Center-based Prospective Study for the Next Generation (JPHC-NEXT). Twelve-day dietary records (DRs) were collected over a one-year period, and food frequency questionnaires (FFQs) were collected twice during the year. Dietary acrylamide intake was estimated from an acrylamide content database. Within-individual variations and between-individual variations were calculated using the random effects model. A linear regression analysis was performed to identify foods with large between-individual variations.

**Results:**

The ratios of within-individual variance to between-individual variation were 3.2 for men and 4.3 for women. Days of DRs required to estimate the usual individual intake within 20% of the true mean intake with 95% confidence were 60 days for men and 66 days for women. Coffee/cocoa, potato, and green tea contributed to between-individual variations, in that order, and seven foods contributed to 93% of the between-individual variation.

**Conclusions:**

Estimating the acrylamide intake using DRs requires an extended data collection period to estimate the intragroup ranking and habitual intake of individuals. Long-term exposure assessments should be based on methods with less potential for measurement errors, such as the use of biomarkers.

## Introduction

Acrylamide is a Group 2A probable human carcinogen [1] that is widely used in industrial applications [2] and is detected in tobacco smoke [3] and food [4]. Acrylamide in food was first discovered in 2002 as a by-product in carbohydrate-rich, heat-processed foods, such as snacks, potato crisps, and bread [4]. The main dietary sources of acrylamide include coffee, bread, and potato chips [5–7]. Considering its potential carcinogenicity, it is critical to assess the effects of long-term exposure to acrylamide.

Methods such as dietary records (DRs), 24-h dietary recalls (24 h-Rs), duplicate methods (DMs), and total diet studies are used to assess dietary acrylamide exposure [5, 8–11]. DRs and 24 h-Rs are commonly used to assess the validity of food frequency questionnaires (FFQs) used to estimate the dietary acrylamide intake [5, 8]. Furthermore, total diet studies have been conducted to assess the risk associated with acrylamide intake [10, 11]. However, because most of these surveys are conducted over a brief period, it is difficult to assess the long-term acrylamide exposure from dietary intake at the individual level. Particularly for nutrients and ingredients that have large within-individual variations, it is necessary to consider whether the estimated intake is representative of the individual’s true intake.

Estimating the true mean intake of acrylamide is important for determining the accurate risk of acrylamide exposure from food. FFQs are well-suited for ranking individuals, but it is difficult to set a threshold because FFQs cannot sufficiently estimate the absolute intake of individuals. However, we can determine the number of days of DRs required to estimate the usual individual intake because long-term DRs allow for the estimation of the individual’s absolute value and variations. An estimation of the true mean intake can be a useful resource for risk assessment and assessing the association with diseases. The estimated value may lead to reference values that can be used for guidelines, as evidence of public health comments, and for individual dietary counseling. To the best of our knowledge, no reported studies have evaluated variations in the estimated within-individual and between-individual dietary acrylamide intakes or investigated the number of days of DRs needed to estimate the individual habitual intake and individual ranking in groups.

FFQs are widely used in large-scale epidemiological studies to determine the effects of long-term dietary acrylamide intake [12, 13]. As with most epidemiologic applications of FFQs, ranking is the primary objective. To rank individuals, FFQs must include foods that have between-individual differences in intake within the group. Although population studies have been performed and reported foods that contribute to the absolute intake of dietary acrylamide [5–7], studies predicting between-individual variations for foods are limited. This study aimed to identify variations in the estimated dietary acrylamide intake of the Japanese population.

## Methods

### Data collection and study participants

Details of the study design and participant characteristics have been described previously [14]. Validation studies of the FFQ were conducted in five areas (Yokote, Saku, Chikusei, Murakami, and Uonuma) according to the protocol of the Japan Public Health Center-based Prospective Study for the Next Generation (JPHC-NEXT) between November 2012 and December 2013 [14]. FFQs were administered twice, with an interval of 1 year. DR reference intake data were obtained from all participants using a consecutive 3-day weighed food record (which included a weekend) at 3-month intervals across all seasons (winter, spring, summer, autumn) [14]. A group of 253 participants completed the 12-day DRs and FFQs. Of the participant group of 253 individuals, this study included 240 participants (98 men and 142 women) 40–74 years of age.

### Database of acrylamide-containing foods

We used an established database of foods that contain acrylamide to estimate the acrylamide intake from the DRs and FFQs [5]. The database was developed using 10 published reports of acrylamide measurements in common Japanese foods [15–24]. The database consists of foods listed in the Standard Tables of Food Composition in Japan, Fifth Revised and Enlarged Edition (5th FCT). Of the 1878 foods listed in 5th FCT, 282 foods contain acrylamide and 1276 foods do not contain acrylamide. The remaining 320 foods do not have appropriate measurements; therefore, they were treated as missing values. Details of the development of the database have been described previously [5]. The database includes heat-processed foods such as bread, biscuits, cookies, and coffee, and home-cooked foods such as stir-fried vegetables, toast, and fried batter [5]. Cooking methods such as deep-frying and baking are considered for the following foods in this database: potatoes, onions, bean sprouts, asparagus, sweet peppers, squash, cabbage, string beans, eggplant, broccoli, podded peas, sweet potatoes, toasted bread, deep-fried batter, and stir-fried rice [5].

### Calculation of dietary acrylamide intake from the dietary records

The study participants recorded menus, food and beverage names, and the amounts consumed (according to weight) in a food diary. Dieticians checked the DRs and coded each food using item numbers from the Standard Tables of Food Composition in Japan - 2010 (FCT 2010) [14]. Acrylamide intake from specific cooked foods not listed in the FCT 2010, such as toasted bread, was calculated by dieticians using the menu to determine how the foods were cooked. The nine cooking methods employed were coded as follows: raw, boiled, deep-fried, deep-fried with batter, baked, stir-fried, steamed, lightly stir-fried, and unclear. The dietary acrylamide intake was calculated by multiplying the amount of each food and its acrylamide-intake value from the database of acrylamide-containing foods. Energy intake was also calculated using the FCT 2010.

### Calculation of dietary acrylamide intake from the food frequency questionnaire

An FFQ was designed to estimate the habitual dietary intake for the previous 1 year [14]. Of the 172 food items considered, the following 36 (21%) were designated as acrylamide-containing foods: rice, miso, beer, baked eel, baked fish paste, fried fish paste, bread, rice cake, Japanese-style confectionary, rice crackers, cakes, biscuits and cookies, chocolates, potato chips, peanuts, fried tofu, roasted and ground beans, sesame, sencha (a type of green tea), bancha (a type of green tea), oolong tea, black tea, coffee, instant coffee, soup, potatoes, sweet potatoes, onions, bean sprouts, asparagus, sweet peppers, squash, cabbage, eggplant, snap beans, and broccoli. For rice, bread, potato, sweet potato, and vegetables (onions, bean sprouts, asparagus, sweet peppers, squash, cabbage, eggplant, snap beans, and broccoli), the acrylamide intake was calculated by considering the cooking methods, because our original FFQ estimated the amount of acrylamide from raw food intake only. Weighted averages were used to estimate the acrylamide intake values of these foods after considering the proportion of acrylamide for each cooking method, which was obtained from DRs of the Japan Public Health Center-based Prospective Study (JPHC Study) [5]. The cooking proportions from the JPHC study were used to limit potential overestimation of validity that could occur by using the cooking proportion calculated from DRs for comparison purposes when examining the validity of the FFQ. The dietary acrylamide intake was estimated by multiplying the acrylamide concentration, consumption frequency, and portion size of each food.

The estimated acrylamide intake value for fried batter was calculated using the participants’ responses to the following question in the original FFQ: “How often do you consume deep-fried foods with batter?” Respondents chose their response from the following six frequency categories: almost never, 1–3 times/month, 1–2 times/week, 3–4 times/week, 5–6 times/week, and daily. Acrylamide intake from these foods was estimated by multiplying the frequency of fried-food consumption with batter by the value of daily acrylamide intake from fried batter calculated from the DRs of the JPHC study [5]. The total acrylamide intake calculated from the FFQ was the sum of the acrylamide intake for each food item and the fried batter.

### Statistical analysis

Mean (with standard deviations [SDs]) and median with 5th and 95th percentile values of dietary acrylamide intake were calculated according to sex. Percentage differences in acrylamide intake according to the DR and FFQ methods were computed using the following formula:

$$ \left.\left(\Big(\mathrm{acrylamide}\ \mathrm{intake}\ \mathrm{from}\ \mathrm{FFQ}-\mathrm{acrylamide}\ \mathrm{intake}\ \mathrm{from}\ \mathrm{DR}\right)/\mathrm{acrylamide}\ \mathrm{intake}\ \mathrm{from}\ \mathrm{DR}\right)\times 100 $$

Data were analyzed using the random effects model:

$$ \mathrm{Nutrient}\ {\mathrm{Y}}_{\mathrm{i}\mathrm{j}\mathrm{k}}=\mu +{\mathrm{participant}}_{\mathrm{i}}+\mathrm{Season}\ {\mathrm{X}}_{\mathrm{i}\mathrm{j}}+\mathrm{day}\ {\mathrm{X}}_{\mathrm{i}\mathrm{j}\mathrm{k}}+{\varepsilon}_{\mathrm{i}\mathrm{j}\mathrm{k}} $$

where μ is the mean of acrylamide intake; participant_i_ is the random variable for variation among subjects; Season X_ij,_ day X_ijk_ represents the random effects of the season and day; and the error term (ε_ijk_) represents the random within-person variance. Using this calculation, acrylamide intake data (level 1) were nested for seasons (level 2), which were nested within participants (level 3). Estimates of within-individual variance $\left({\hat{\sigma}}_w^2\right)$ and between-individual variance $\left({\hat{\sigma}}_b^2\right)$ were calculated by setting mean squares (MSs) equal to their expected values. Variances were estimated using the Mixed procedure in SAS (version 9.4; SAS Institute Inc., Cary, NC, USA). We used untransformed data to analyze within-individual and between-individual variations because a previous study suggested that transformed data are likely to underestimate the number of days required for ranking individuals in a group [25].

We also calculated the number of days of DRs needed to estimate an individual’s habitual intake with 95% confidence within a specified percentage deviation using the following formula [26, 27]:

$$ \mathrm{D}={\left({\mathrm{Z}}_{\upalpha}{\mathrm{CV}}_{\mathrm{w}}/\mathrm{E}\right)}^2 $$

where D is the number of days needed per person; Z_α_ is 1.96; CV_w_ is the within-individual coefficient of variation; $\frac{\sqrt{{\hat{\sigma}}_w^2}}{\mathrm{mean}\ \mathrm{acrylamide}\ \mathrm{intake}}\times 100$; and E is the specific degree of error as a percentage of long-term habitual intake (10% or 20%).

The number of days (D) needed to obtain a given unobservable correlation between the observed and true mean intake was calculated using the following formula [26]:

$$ \mathrm{D}=\frac{{\mathrm{r}}^2}{1-{\mathrm{r}}^2}\times \frac{{\hat{\sigma}}_w^2}{{\hat{\sigma}}_b^2} $$

where r is the unobservable correlation between the observed and true mean nutrient intakes of an individual during the period of observation and D indicates days. The chosen value of r is dependent on the degree of acceptable misclassification [26]. We set the r values to 0.9, 0.8, and 0.5 to estimate the number of days required to rank individuals.

A linear regression analysis with stepwise selection was used to identify foods that contributed to between-individual variations, with acrylamide intake from each food item as the explanatory variable and the total acrylamide intake according to the DRs as the response variables. A partial R-squared model value was developed for the selected food items.

The following analysis was conducted to determine the validity and reproducibility of the FFQ and the contribution of each food to the total acrylamide intake. The results are summarized in Additional file 1: Table S1 and Additional file 2: Table S2. We used log-transformed data for energy and acrylamide intake for this analysis. Spearman’s rank correlation coefficients for DR and FFQ estimations were computed for crude and energy-adjusted values, and the energy was adjusted using the residual method. Additionally, deattenuated correlation coefficients were calculated as the correlation coefficients for DR and the FFQ estimations that were attenuated by individual variations in daily intake. Deattenuation was calculated using the following formula:

$$ \mathrm{Deattenuated}\ \mathrm{correlation}\ \mathrm{coefficient}=\mathrm{energy}-\mathrm{adjusted}\ \mathrm{correlation}\ \mathrm{coefficient}\times \sqrt{1+\frac{\lambda }{n}} $$

where λ is the ratio of within-individual variations and between-individual variations of DRs, and n is the number of DRs for each participant (12 days). For the cross-classification analysis, energy-adjusted acrylamide intake from DRs and the FFQ were categorized into quintiles, and the proportion of participants among the same, adjacent, and opposite categories were calculated using both quintile numbers. In addition, weighted kappa coefficients were computed. The contribution of each food to the total acrylamide intake was computed as the percentage of acrylamide intake from each food for the total amount of acrylamide intake from DR data. In addition, Spearman’s correlation coefficients for the FFQ and DR estimations were calculated as FFQ validity. All analyses were performed using SAS (version 9.4).

## Results

### Participant characteristics

Participants in this study had mean ages of 57.4 years (men) and 57.0 years (women). The mean body mass index (BMI) values were 23.7 kg/m^2^ for men and 22.8 kg/m^2^ for women. The percentages of current smokers were 26.5% for men and 2.1% for women [14]. Participant characteristics according to acrylamide intake are shown in Table 1. The mean ± SD dietary acrylamide intake values for men were 4.7 ± 1.3, 8.8 ± 1.2, and 15.8 ± 4.3 μg/day for the lowest, middle, and highest tertile of dietary acrylamide intake, respectively. For women, these values were 5.7 ± 1.3, 9.3 ± 1.2, and 15.6 ± 5.2 μg/day for the lowest, middle, and highest tertile of dietary acrylamide intake, respectively. The coffee intake (g/day) increased linearly from the lowest to the highest tertile for both men and women. Table 2 shows the mean, standard deviation, median, and the 5th–95th percentiles of acrylamide intakes estimated from the DRs and FFQs. The mean acrylamide intake values estimated from DRs were 0.15 μg/kg body weight/day for men and 0.19 μg/kg body weight/day for women, and the 95th percentile values were 0.27 μg/kg body weight/day for men and 0.32 μg/kg body weight/day for women. The percentage differences in the means estimated by DR and FFQ were underestimated by 7 and 5% for body weight of men and women, respectively.

**Table 1:** Characteristics of the participants

	Tertiles of acrylamide intake		
	Lowest	Middle	Highest
	T1 Mean ± SD or %	T2 Mean ± SD or %	T3 Mean ± SD or %
Men No. of participants	32	33	33
Acrylamide intake			
Mean (μg/day)^a^	4.7 **±** 1.3	8.8 **±** 1.2	15.8 **±** 4.3
Median (μg/day) ^a^	4.9	8.9	14.6
Age			
Mean (years)	60 **±** 8	55 **±** 8	57 **±** 10
Median (years)	62.0	56.0	57.0
Body mass index			
Mean (kg/m^2^)	22.9 **±** 2.2	24.0 **±** 3.2	24.1 **±** 2.9
Median (kg/m^2^)	23.2	24.5	24.0
Smoking (n,%)			
Never smoker	7 21.9	10 30.3	6 18.2
Ex-smoker	18 56.3	13 39.4	18 54.5
Current smoker	7 21.9	10 30.3	9 27.3
Education (n,%)			
Junior high school	6 18.8	2 6.1	1 3.0
High school	13 40.6	14 42.4	10 30.3
Some college	7 21.9	10 30.3	8 24.2
College or more	6 18.8	7 21.2	14 42.4
Dietary intake Mean			
Energy intake (kcal) ^a^	2082 **±** 363	2287 **±** 383	2570 **±** 456
Coffee (g/day) ^a^	32 **±** 42	141 **±** 109	192 **±** 199
Green tea (g/day) ^a^	333 **±** 312	267 **±** 194	437 **±** 465
Potato (g/day) ^a^	18 **±** 12	18 **±** 15	33 **±** 27
Biscuit and cookies (g/day) ^a^	0.5 **±** 1	1 **±** 2	3 **±** 3
Women No. of participants	47	48	47
Acrylamide intake			
Mean (μg/day) ^a^	5.7 **±** 1.3	9.3 **±** 1.2	15.6 **±** 5.2
Median (μg/day) ^a^	6.1	9.3	13.9
Age			
Mean (years)	58 **±** 9	57 **±** 8	56 **±** 9
Median (years)	57	59	56
Body mass index			
Mean (kg/m^2^)	23.5 **±** 3.6	22.2 **±** 2.5	22.7 **±** 3.0
Median (kg/m^2^)	23.0	22.3	22.3
Smoking (n,%)			
Never smoker	44 93.6	47 97.9	41 87.2
Ex-smoker	2 4.3	0 0.0	5 10.6
	Tertiles of acrylamide intake		
	Lowest	Middle	Highest
	T1 Mean ± SD or %	T2 Mean ± SD or %	T3 Mean ± SD or %
Current smoker	1 2.1	1 2.1	1 2.1
Education (n,%)			
Junior high school	6 12.8	3 6.3	4 8.5
High school	20 42.6	17 35.4	14 29.8
Some college	16 34.0	27 56.3	22 46.8
College or more	4 8.5	1 2.1	6 12.8
Other	1 2.1	0 0.0	1 2.1
Dietary intake Mean			
Energy intake (kcal)	1672 **±** 216	1775 **±** 246	1969 **±** 371
Coffee (g/day) ^a^	40 **±** 41	92 **±** 101	224 **±** 257
Green tea (g/day) ^a^	390 **±** 276	382 **±** 308	497 **±** 397
Potato (g/day) ^a^	16 **±** 9	18 **±** 12	23 **±** 21
Biscuit and cookies (g/day) ^a^	1 **±** 2	3 **±** 3	4 **±** 5

^a^ Crude intake

**Table 2:** Comparison of acrylamide intake mean values from dietary records and food frequency questionnaires

	DR					FFQ					%^a^
	Mean	(SD)	Median	(5^th^percentile, 95^th^percentile)		Mean	(SD)	Median	(5^th^percentile, 95^th^percentile)		
*Crude acrylamide intake (μg/day)*											
Men (*n* = 98)	9.80	(5.34)	8.87	(3.54,	21.24)	9.27	(5.14)	8.44	(3.20,	22.86)	-5
Women (*n* = 142)	10.21	(5.15)	9.33	(4.14,	17.60)	9.98	(4.88)	9.39	(3.80,	18.97)	-2
All (*n* = 240)	10.04	(5.22)	9.00	(4.00,	19.16)	9.69	(4.99)	8.99	(3.60,	19.50)	−3
*Crude acrylamide intake (μg/kg body weight/day)*											
Men (n = 98)	0.15	(0.08)	0.13	(0.05,	0.27)	0.14	(0.08)	0.12	(0.05,	0.33)	−7
Women (n = 142)	0.19	(0.10)	0.17	(0.07,	0.32)	0.18	(0.09)	0.17	(0.07,	0.36)	−5
All (n = 240)	0.17	(0.09)	0.15	(0.06,	0.31)	0.16	(0.09)	0.15	(0.06,	0.34)	−6

*DR* dietary record, *FFQ* food frequency questionnaire for validation analysis, *SD* standard deviation

^a^ Percentage differences (%) were calculated from the following formula: (“mean FFQ” − “mean DR”)/ “mean DR” × 100

### Between-individual and within-individual variations

Table 3 shows the relative contributions of between-individual and within-individual variances to the total variance in acrylamide intake and the coefficients of within-individual variance (CV_w_) and between-individual variance (CV_b_). In addition, Table 3 includes the number of days of DRs required to estimate the individual’s habitual intake within 10 and 20% of their true mean intake. The ratios of within-individual variation to between-individual variation were 3.2 for men and 4.3 for women. Days of DRs necessary for estimating the true intake within 20% were 60 days for men and 66 days in women. Days required to ensure an r value of 0.8 for ranking individuals were 6 days for men and 8 days for women. In the sensitivity analysis, ratios of within-individual variance to between-individual variance and the number of days were calculated by the log-transformed data; the ratios of within-individual variation to between-individual variance were 1.9 for men and 2.8 for women. Therefore, the number of days for ranking was underestimated as a previous study suggested [25]. In the additional sensitivity analysis, we calculated the ratios of within-individual variance to between-individual variance using 12-day independent records without considering the data of consecutive 3-day records, and these variance ratios were almost the same.

**Table 3:** Relative contributions of within- and between-individual variance values, coefficients of within-individual variance (CV_w_) and between-individual variance (CV_b_), the number of days required for collecting food records necessary to estimate the true intake within 10 and 20% of the true mean, and the number of days required to ensure r = 0.9, 0.8 and 0.5 between observed and true mean intake

	Percentage contributions of variance components within-individual	Percentage contributions of variance components between-individual	VR	Mean intake (μg/day)	CV_w_^a^ (%)	CV_b_^b^ (%)	D_1_ 10%(days) ^c^	D_2_ 20%(days) ^c^	D_3_ r = 0.9 (days)^d^	D_4_ r = 0.8 (days)^d^	D_5_ r = 0.5 (days)^d^
Men (n = 98)	76.3	23.7	3.2	9.80	79.1	44.0	240	60	14	6	1
Women (n = 142)	81.2	18.8	4.3	10.21	83.0	39.9	265	66	18	8	1
All (n = 240)	79.8	20.2	3.9	10.04	81.5	41.5	255	64	16	7	1

CV_w_, coefficient of within-individual variation; CV_b_, coefficient of between-individual variation; VR, ratio of within- to between- individual variance $\left(\frac{{\hat{\sigma}}_w^2}{{\hat{\sigma}}_b^2}\right)$

^a^$$ \mathrm{CVw}=\frac{\sqrt{{\hat{\sigma}}_w^2}}{\mathrm{mean}\ \mathrm{acrylamide}\ \mathrm{intake}}\times 100 $$

^b^$$ \mathrm{CVb}=\frac{\sqrt{{\hat{\sigma}}_b^2}}{\mathrm{mean}\ \mathrm{acrylamide}\ \mathrm{intake}}\times 100 $$

^c^Number of days needed to lie within specified % of the true means: D_1.2_ = (Z_α_CV_w_/E)^2^, where D = number of days needed per person, Z_α_ = normal deviate (1.96), E = specific error admitted as a percentage of the true usual intake; 10% or 20%

^d^Number of days required to ensure r = 0.9 or 0.8 or 0.5 between observed and true mean intake: ${\mathrm{D}}_{3,4,5}=\left[\frac{r^2}{\left(1-{r}^2\right)}\right]\times \mathrm{VR}$, where r = the unobservable correlation coefficient between the observed and true mean nutrient intakes of the individual

Table 4 shows the foods that best predicted the between-individual variations in acrylamide intake. Foods that contributed to the between-individual variations were, from highest to lowest contribution, as follows: coffee/cocoa, potatoes, green tea, sweet potatoes, and biscuits/cookies. The top seven foods accounted for approximately 93% of the total variation.

**Table 4:** Foods with the highest variations in the estimated between-individual variation in dietary acrylamide from dietary records

	Foods	Partial R-Square ^a^	Cumulative R-Square ^a^
1	Coffee and Cocoas	0.333	0.333
2	Potatoes	0.212	0.545
3	Green teas	0.156	0.701
4	Sweet potatoes	0.098	0.799
5	Biscuits	0.060	0.859
6	Traditional dry confectionary	0.039	0.898
7	Snacks (Potato chips)	0.035	0.933
8	Beans sprouts	0.017	0.950
9	Chocolates	0.010	0.960
10	Podded pods	0.009	0.969

^a^ Foods were selected by stepwise regression analysis using data from DR. Partial and cumulative R-Square values were calculated in the process of performing the regression analysis

The validity of the estimated acrylamide intake determined by using the FFQ indicated that the energy-adjusted correlation coefficients were 0.34 for men and 0.28 for women, and that the deattenuated correlation coefficients were 0.39 for men and 0.33 for women. The reproducibility of the FFQ indicated that the energy-adjusted correlation coefficients were 0.62 for men and 0.65 for women (Additional file 1: Table S1). In the sensitivity analysis, the acrylamide intake calculated using the cooking proportion from the DRs was compared to the validity of the FFQ, and the Spearman’s correlation coefficients for crude and energy-adjusted intake were unchanged. The food groups from the DRs that contributed most the acrylamide intake were beverages, confections, vegetables, potatoes and other starches, and cereals (Additional file 2: Table S2).

## Discussion

We demonstrated that estimating the habitual dietary acrylamide intake from DRs requires an extended data collection period because of the large within-individual variation in dietary acrylamide intake. Furthermore, the between-individual variation was largely accounted for by seven foods.

Although there have been no reports of the within-individual and between-individual variations of acrylamide intake according to DRs, many other nutrients tend to have larger within-individual variations than between-individual variations; for example, the variance ratios are 1.0–2.2 for energy, 1.6–3.1 for protein, and 3.2–5.4 for fat [25, 28–31]. For reference, the variance ratios determined by this study were approximately 1 for energy, 1.3 for protein, and 1.8 for fat. It was also revealed that confections and potatoes greatly contributed to the absolute intake of acrylamide. As a Japanese study previously reported, there is large within-individual variation in the consumption of these foods [28]. Therefore, we think that these foods are likely to be consumed sporadically, which explains their large within-individual variations. The within-individual variation of non-smokers was also reported to be high in a previous study that examined within-individual variation using hemoglobin adducts, which are biomarkers of acrylamide consumption [32], and our results are consistent with those. As such, it is necessary to choose a precise method of exposure assessment based on the large within-individual variation.

Moreover, the within-individual and between-individual variations of acrylamide intake estimated by DRs showed that estimations of the intragroup ranking and the individual’s habitual intake required data collection for an extended period. An analysis using 24 h-Rs with a correlation of 0.8 showed the following relationships between the number of days and variation ratios: approximately 5 days for a variation ratio of 1; 10 days for a variation ratio of 3; and 20 days for a variation ratio of 5; in other words, when the variation ratio is larger, more days of DRs are needed [31]. As shown by our results, when the variation ratio and/or within-individual variation is large, then the estimation of acrylamide intake using short-term dietary surveys may lead to a misclassification of rankings and may not represent an individual’s habitual intake. This error distorts the correlation coefficient of validation studies and reduces the strength of association with disease in epidemiological studies [27]. In fact, an FFQ validation study using 24 h-Rs suggested that the main reason for the low correlation coefficient was caused by a large within-individual variation in 24 h-Rs [8]. Therefore, methods that estimate acrylamide intake during short periods of time should be cautiously reviewed considering the influence of within-individual variation.

Foods ranked as high for their predicted between-individual variation were also ranked high for their contribution to the absolute intake of acrylamide. This may be because the intake of these foods is highly dependent on individual preference and they contribute significantly to both individual intake and between-individual variation. Although snacks did not contribute significantly to the absolute intake, their ranking among foods contributing to between-individual variation was high. Therefore, it is conceivable that it is essential to analyze snacks to detecting differences in acrylamide intake among individuals. Collectively, our results suggest that it is possible to rank acrylamide intake according to several kinds of foods.

Furthermore, the mean intake in our study was lower than that observed in Western populations, and it was similar to that of the previous Monte Carlo simulation studies of Japanese populations (mean, 0.166 μg/kg body weight/day; 95th percentile, 0.261 μg/kg body weight/day) [33]. In the current study, the mean intake was 0.05 μg/kg body weight/day higher than that of the previous JPHC study [5]. This was primarily due to increases in contribution associated with the intake of coffee/cocoa and confections. The acrylamide intake values of traditional fresh and semi-dry confections in the current study were half that reported by the JPHC study [5], but intake values of chocolate and potato chips were more than 50% higher. Our results indicate the prime importance of evaluating exposure from the viewpoint of risk assessment to mitigate potential health challenges.

To the best of our knowledge, this is the first study to elucidate the within-individual variation and between-individual variation in dietary acrylamide intake. The key contribution of this study was that it assessed the within-individual variation of acrylamide intake, which must be considered as a measurement error that potentially contributes to erroneous findings. These findings highlight the need to use biomarkers to appropriately estimate long-term acrylamide exposure because biomarkers are thought to have fewer measurement errors than other measurement methods. The between-individual variation results of our study also show that it is possible to rank acrylamide intake based on several types of foods, thus implying that a simpler questionnaire could be developed that specifically targets the acrylamide intake of the Japanese population in the future.

There were certain limitations to this study. First, the results of this study depended on the accuracy of acrylamide content database. The database includes approximately 50% of foods that may contain acrylamide. If there are large within-individual variations in foods that are not included in the database, then it may take more days to rank individuals and to estimate habitual intake from DRs. Second, the study did not include individuals 39 years or younger or individuals 75 years or older; therefore, the applicability of the results to all age groups remains uncertain. For other nutrients, within-individual variations tend to be larger in younger people than in older people [30], suggesting that more days may be required to accurately estimate the habitual intake of dietary acrylamide from the DRs of a young population. Finally, a comparison of dietary acrylamide intake and biological markers, such as glycidamide and acrylamide hemoglobin adducts, was not possible. Assessing the within-individual variability and between-individual variability of acrylamide intake from both diet and biomarkers would help to address the current challenges, such as determining the cause of the low correlation between the 24 h-Rs, FFQs, and biomarkers [8]. Importantly, a better estimation of acrylamide exposure would enhance the reliability of evidence in epidemiological studies.

## Conclusion

Overall, the study results revealed that the estimation of habitual acrylamide intake and the ranking within the group according to DRs require an extended data collection period because of large within-individual variations. The estimation of acrylamide intake using short-term dietary surveys may lead to the misclassification of rankings and may not represent an individual’s habitual intake. Therefore, we conclude that assessing the long-term exposure based on methods that are unaffected by measurement error such as those associated with biomarkers is required, and that a repeated assessment of these biomarkers would be beneficial.

## Supplementary information

**Additional file 1 **: **Table S1.** Evaluation of the relationships between dietary records, food frequency questionnaires used for the validation analysis, and food frequency questionnaires.

**Additional file 2 **: **Table S2.** Contribution of food groups to total acrylamide intake estimated using dietary records.

## Data Availability

We cannot publicly provide individual data due to participant privacy, according to ethical guidelines in Japan. Additionally, the informed consent we obtained does not include a provision for publicly sharing data. The datasets used and/or analysed during the current study are available from the corresponding author on reasonable request.

## Abbreviations

24 h-Rs: 24-h dietary recalls
DM: Duplicate method
DR: Dietary record
FCT 2010: The Standard Tables of Food Composition in Japan 2010
FFQ: Food frequency questionnaire
JPHC: The Japan Public Health Center-based Prospective Study
JPHC-NEXT: The Japan Public Health Center-based Prospective Study for the Next Generation
