# Supplementary material for: Variations in the estimated intake of acrylamide from food in the Japanese population
Source: Nutr J. 2020 Feb 21;19:17. doi: 10.1186/s12937-020-00534-y (PMC7035741; doi:10.1186/s12937-020-00534-y)
Supplement: Supplementary file 1 — Additional file 1 : Table S1. Evaluation of the relationships between dietary records, food frequency questionnaires used for the validation analysis, and food frequency questionnaires. [file 12937_2020_534_MOESM1_ESM.docx]

**Additional file 1: Table S1.**

Evaluation of the relationships between dietary records, food frequency questionnaires used for the validation analysis, and food frequency questionnaires

|  | Validity^a^ | | |  | Cross-classiﬁcation^b^ | | | |  | Reproducibility^c^ | |
| --- | --- | --- | --- | --- | --- | --- | --- | --- | --- | --- | --- |
|  | Crude | Energy-adjusted | Deattenuated^d^ |  | Same  category | Same and adjacent  category | Extreme  category | Weighted κ  coefficient |  | Crude | Energy-  adjusted |
| Men (n=98) | 0.39 | 0.34 | 0.39 |  | 29 | 63 | 3 | 0.83 |  | 0.67 | 0.62 |
| Women (n=142) | 0.25 | 0.28 | 0.33 |  | 20 | 61 | 4 | 0.81 |  | 0.74 | 0.65 |

DR, dietary record; FFQ_V, food frequency questionnaire for validation analysis.

^a^ Spearman’s correlation coefficients between DRs and the FFQ_V.

^b^ Percentages were presented based on the cross-classiﬁcation by quintile between DRs and the FFQ_V.

^c^ Spearman’s correlation coefficients between the two FFQs.

^d^Deattenuated CC = Energy-adjusted CC×$\sqrt{1+(\lambda/ｎ)}$ , where λ is the ratio of within- to between-individual variance and n is the number of DRs.
