# Supplementary material for: Variations in the estimated intake of acrylamide from food in the Japanese population
Source: Nutr J. 2020 Feb 21;19:17. doi: 10.1186/s12937-020-00534-y (PMC7035741; doi:10.1186/s12937-020-00534-y)
Supplement: Supplementary file 2 — Additional file 2 : Table S2. Contribution of food groups to total acrylamide intake estimated using dietary records. [file 12937_2020_534_MOESM2_ESM.docx]

**Additional file 2: Table S2.**

Contribution of food groups to total acrylamide intake estimated using dietary records

| Food group name | | Proportion (%) | Number of assigned foods | Top 5 contributing foods (%) | | | | | | | | | | | |
| --- | --- | --- | --- | --- | --- | --- | --- | --- | --- | --- | --- | --- | --- | --- | --- |
|  |  |  |  | 1 | | 2 | | | 3 | | 4 | | | 5 | |
| Beverages | 40.8 | | 22 | Coffees and cocoas | (24.8) | Green teas | (11.7) | Fermented alcoholic beverages | | (2.4) | | Mugi-cha | (1.5) | Fermented teas | (0.5) |
| Confectionary | 19.9 | | 74 | Traditional dry confectionary | (5.8) | Biscuits and cookies | (5.0) | Chocolates | | (3.7) | | Snacks | (2.4) | Cakes, buns and pastries | (1.8) |
| Vegetables | 14.2 | | 33 | Bean sprouts | (4.3) | Sweet peppers | (3.2) | Onions | | (2.3) | | Cabbages | (1.2) | Eggplants | (1.1) |
| Potatoes and Starches | 10.6 | | 7 | Potatoes | (6.9) | Sweet potatoes | (3.6) |  | |  | |  |  |  |  |
| Cereals | 6.5 | | 46 | Rice | (2.7) | Noodles dried by frying | (2.0) | Breads | | (1.5) | | Bread crumbs | (0.2) | Cornflakes | (0.1) |
| Seasoning and Spices | 2.7 | | 23 | Roux | (1.9) | Miso | (0.4) | Soy sauce | | (0.3) | |  |  |  |  |
| Nuts and Seeds | 2.1 | | 13 | Sesame seeds | (1.1) | Walnuts | (0.4) | Peanuts | | (0.4) | | Cashew nuts | (0.1) | Almonds | (0.05) |
| Fish and Shellfishes | 1.1 | | 33 | Baked fish | (0.8) | Fish paste products (baked or fried) | (0.3) |  | |  | |  |  |  |  |
| Fruits | 0.8 | | 7 | Dried fruits | (0.8) |  |  |  | |  | |  |  |  |  |
| Pulses | 0.7 | | 10 | Tofu (baked or fried) | (0.4) | Roasted and ground soybeans | (0.2) | Beans cooked with sugar and salt | | (0.04) | |  |  |  |  |
| Sugars and Sweeteners | 0.4 | | 2 | Brown sugar | (0.4) |  |  |  | |  | |  |  |  |  |
